# Maintenance rucaparib after first-line platinum-based chemotherapy in **HER2-negative** advanced oesophagogastric adenocarcinoma: results from the PLATFORM study

**DOI:** 10.1016/j.esmoop.2026.108315

**Published:** 2026-08-10

**Authors:** A. Gordon, C. Fong, L. Satchwell, S. Cromarty, K. Piadel, A. Tran, R. Begum, A. Woodward, B. Leamon, O. Zhitkov, M. Terlizzo, M. Hubank, P. Proszek, G. Pietka, E. Peat, E. Cartwright, M. Davidson, P. Das, S. Sothi, R. Herbertson, S. Darby, T. Waddell, A. Bradshaw, S. Rao, N. Starling, I. Chau, D. Cunningham

**Affiliations:** 1 Gastrointestinal Unit, Department of Medicine, Royal Marsden Hospital, London & Sutton, UK; 2 Research Data & Statistics Unit, Royal Marsden Clinical Trials Unit, Royal Marsden Hospital, London & Sutton, UK; 3 Department of Histopathology, Royal Marsden Hospital, London, UK; 4 Department of Molecular Diagnostics, Royal Marsden Hospital, Sutton, UK; 5 The Institute of Cancer Research, Sutton, UK; 6 Department of Oncology, Royal Derby Hospital, University of Derby and Burton NHS Foundation Trust, Derby, UK; 7 Department of Oncology, University Hospital Coventry, University Hospitals Coventry and Warwickshire NHS Trust, Coventry, UK; 8 Sussex Cancer Centre, Royal Sussex County Hospital, University Hospitals Sussex NHS Foundation Trust, Brighton, UK; 9 Weston Park Hospital, Sheffield Teaching Hospitals NHS Foundation Trust, Sheffield, UK; 10 Department of Medical Oncology, The Christie NHS Foundation Trust, Manchester, UK; 11 Cancer Services, Newcastle upon Tyne Hospitals NHS Foundation Trust, Newcastle upon Tyne, UK

**Keywords:** DNA damage response, gastric cancer, oesophageal cancer, PARP inhibitors

## Abstract

**Background:**

PLATFORM is a prospective, open-label, multicentre, adaptive phase II trial assessing maintenance therapy in patients with advanced HER2-negative oesophagogastric adenocarcinoma after platinum-based first-line chemotherapy.

**Patient and methods:**

After 18 weeks of platinum-based chemotherapy, patients with response or stable disease were randomised to surveillance (4 weekly visits) or rucaparib 600 mg tablet twice daily every 28 days. The primary endpoint was progression-free survival (PFS) (prespecified *P* value of 0.025). Secondary endpoints were safety and overall survival (OS). Exploratory translational analyses of homologous recombination deficiency (HRD) and somatic gene profiling are also reported.

**Results:**

A total of 125 patients were randomised (surveillance: 62, rucaparib: 63). The median follow-up was 41 months. Median PFS was 2.8 months for surveillance and 4.2 months for rucaparib [hazard ratio (HR) 0.70, 95% confidence interval (CI) 0.49-1.02, *P* = 0.031]. There was no difference in OS (HR 1.15, 95% CI 0.77-1.72, *P* = 0.247). Grade ≥3 adverse events (AEs) occurred in 23% of surveillance and 32% of rucaparib patients. Treatment-related AEs were reported in 84% of patients in the rucaparib arm; 22% of which were grade 3-4. Most frequent grade 3-4 AEs were anaemia, fatigue, infection, and neutropenia. Adequate tissue for HRD analysis was available in 51/125 (40.8%) patients (surveillance: 20, rucaparib: 31). Five patients (9.8%) were HRD-positive, 41 (80.4%) HRD-negative, and 5 (9.8%) had inconclusive results. Among HRD-positive patients, four were treated with rucaparib and had PFS durations of 2.6, 3.0, 8.3, and 10.6 months, respectively. *TP53* was the most frequently detected somatic alteration, followed by *SMARCA4* and *ARID1A*.

**Conclusion:**

Compared with surveillance, maintenance rucaparib following first-line chemotherapy in patients with advanced HER2-negative OGA did not significantly improve PFS with no observed difference in OS. Translational analyses were limited by small sample size, and no clear associations with clinical outcomes were identified.

## Highlights

• Maintenance rucaparib did not significantly improve PFS compared with surveillance.
• Rucaparib was associated with higher rates of grade ≥3 AEs compared with surveillance.
• No definitive association was observed between HRD status or somatic alterations and clinical outcomes.

## Introduction

Oesophagogastric cancer represents a major global health burden and remains a leading cause of cancer-related mortality, largely as most patients present with advanced, inoperable disease. In biomarker unselected populations, palliative chemotherapy is associated with a median overall survival (OS) of <12 months.^1^, ^2^, ^3^, ^4^ The integration of targeted therapies into first-line treatment has extended median OS up to 18 months in biomarker-selected subgroups, including those expressing HER-2, programmed death-ligand 1 (PD-L1), and CLDN18.2.^5^, ^6^, ^7^, ^8^, ^9^ Despite these advances, outcomes remain suboptimal for many patients and there is a continued need to identify additional predictive biomarkers to refine patient selection and optimise current treatment strategies.

Oesophagogastric adenocarcinoma (OGA) is characterised by high genomic instability, largely driven by defects in deoxyribonucleic acid (DNA) damage response (DDR) pathways.^10^^,^^11^ Effective DDR is crucial for maintaining genomic integrity, and failure to repair DNA damage leads to genomic instability and the persistent accumulation of mutations that promote malignant transformation and tumourigenesis.^12^^,^^13^ These vulnerabilities create a therapeutic opportunity to target defective DDR mechanisms.

The poly(ADP-ribose) polymerase (PARP) proteins comprise a family of 17 enzymes involved in DNA transcription, DDR, genomic stability maintenance, cell cycle regulation, and cell death.^14^ PARP1 and PARP2 are central to the repair of single-strand DNA breaks through the base excision repair pathway. PARP inhibition leads to the accumulation of single-strand breaks, which are converted to double-strand breaks during DNA replication.^15^ These lesions are normally repaired through homologous recombination, a complex DNA repair pathway dependent on *BRCA1* and *BRCA2*. Loss-of-function alterations in *BRCA1/2* can lead to homologous recombination deficiency (HRD), promoting genomic instability.^16^ In HRD cells, PARP inhibition leads to irreparable DNA damage and cell death through synthetic lethality, a mechanism that underpins the clinical activity of PARP inhibitors in HRD-associated tumours, particularly those harbouring *BRCA1/2* mutations.^17^, ^18^, ^19^

PARP inhibitors are established in ovarian, breast, prostate, and pancreatic cancers.^20^, ^21^, ^22^, ^23^, ^24^ Clinical benefit has been most remarkable in the maintenance setting, particularly in platinum-sensitive ovarian cancer, where significant progression-free survival (PFS) and OS benefits have been demonstrated.^21^^,^^25^, ^26^, ^27^, ^28^, ^29^, ^30^, ^31^ Maintenance PARP inhibition has also improved PFS in BRCA-mutated pancreatic cancer.^22^ Rucaparib has demonstrated efficacy in metastatic ovarian and prostate cancers and is licensed as maintenance therapy for platinum-sensitive ovarian cancer irrespective of BRCA or HRD status.^30^^,^^31^ It is also approved for BRCA1/2-mutated metastatic castration-resistant prostate cancer.^20^^,^^21^

Sensitivity to platinum chemotherapy has been associated with increased responsiveness to PARP inhibition, likely reflecting shared dependence on defective DDR pathways.^32^ Given the central role of platinum chemotherapy in the first-line treatment of advanced OGA, and the potential molecular parallels with other platinum-sensitive cancers, maintenance PARP inhibition represents a biologically rational therapeutic strategy.

On this basis, rucaparib was incorporated into the PLATFORM trial. PLATFORM (PLAnning Treatment for Oesophago-gastric Cancer: a Randomised Maintenance Therapy Trial) is an adaptive, multiarm, phase II trial investigating various maintenance therapies after first-line platinum-based chemotherapy in patients with advanced, HER2-negative OGA. The original trial design randomised patients to surveillance, capecitabine, or durvalumab. Subsequent protocol amendments introduced rucaparib and capecitabine plus ramucirumab (cape-ram) arms, resulting in a final randomisation of 1 : 1 : 1 : 1 : 1 across the five arms. Each arm was compared independently against surveillance (Supplementary Figure S1, available at https://doi.org/10.1016/j.esmoop.2026.108315). Accrual to the durvalumab, rucaparib, and cape-ram arms was discontinued early following withdrawal of industry support, whereas the capecitabine arm completed full recruitment in May 2024. Results from the capecitabine, durvalumab, and cape-ram analyses have been reported previously.^33^, ^34^, ^35^

Recruitment to the rucaparib arm ceased in November 2022 after Clovis Oncology withdrew industry support. As a result, full recruitment was not achieved. This occurred before the planned interim futility analysis, although the prespecified number of events for that analysis had already been reached. Here, we report the primary results of the randomised comparison between maintenance rucaparib and active surveillance, together with exploratory translational results.

## Patients and methods

This has been published previously.^33^^,^^34^

### Participants

Eligible patients were adults (age ≥18 years) with inoperable locally advanced or metastatic adenocarcinoma of the oesophagus, gastro-oesophageal junction (GOJ) or stomach, and had achieved stable disease (SD) or response on computed tomography (CT) after first-line fluoropyrimidine-platinum chemotherapy. The initial protocol mandated completion of at least six cycles of investigators’ choice of first-line fluoropyrimidine-platinum chemotherapy. This was later limited to 18 weeks’ duration and the regimens were limited to capecitabine and oxaliplatin (CAPOX), cisplatin and capecitabine (CX), or 5-fluorouracil and oxaliplatin (FOLFOX). This protocol amendment was instituted during the initial recruitment phase of PLATFORM as we observed a high attrition rate of over 50% of registered patients not proceeding to randomisation secondary to disease progression after eight cycles (24 weeks) of chemotherapy. Patients who could not tolerate 18 weeks of first-line fluoropyrimidine-platinum chemotherapy were excluded from randomisation. Patients were required to have HER2-negative disease, performance status 0-2, and adequate organ function. Prior exposure to PARP inhibitors was an exclusion criterion. Measurable disease was not required for trial entry. PD-L1 and mismatch repair were not eligibility criteria as it was not standard of care when the protocol was established. However, this was assessed in the experiment arm with durvalumab.^34^

### Trial design

Patients were recruited across 46 centres in the United Kingdom. The adaptive study design allowed early closure of ineffective treatment arms and the addition of new experimental arms. Patients were registered before or during first-line chemotherapy. Those who achieved disease control after 18 weeks of chemotherapy and met eligibility criteria were randomised to surveillance (control arm) or one of the maintenance arms (Supplementary Figure S1, available at https://doi.org/10.1016/j.esmoop.2026.108315). Randomisation was carried out at the Institute of Cancer Research Clinical Trials and Statistics Unit by random permuted blocks. A preplanned interim futility analysis was scheduled after recruitment of 61 radiologically evaluable patients at 12 weeks. Although this milestone was reached, the rucaparib arm closed due to withdrawal of industry support.

### Treatment

Patients randomised to surveillance were reviewed every 28 days and patients assigned to rucaparib received an oral dose of 600 mg twice daily continuously of a 28-day cycle to progression, death, or toxicity. Rucaparib was provided by Clovis Oncology at no cost.

### Study endpoints

The primary endpoint was PFS, defined as time from randomisation to radiological progression by RECIST 1.1, clinical progression, or death. Secondary endpoints included OS, defined as time from randomisation to death, objective response rate (ORR), and safety. Patient-reported outcome was not collected as this study was designed to be a signal-seeking adaptive randomised phase II study with any observed benefits to be confirmed in subsequent phase III trials.

An exploratory analysis of PFS and OS was conducted based on HRD status. We hypothesised that HRD-positive tumours were predictive of improved survival compared with HRD-negative tumours when treated with rucaparib.

### Sample size and statistical analyses

To detect a PFS HR of 0.7 with 80% power and a one-sided alpha of 0.025, 154 patients per arm were required, assuming a 10% dropout rate and median PFS of 6 months in the surveillance arm (data from REAL-2).^3^ A total of 248 PFS events were needed for definitive analysis, assuming a 3-year accrual and maximum 4-year follow-up. A futility analysis based on 12-week progression-free rate (PFR) was triggered once 61 evaluable patients per interventional arm underwent a restaging CT scan post-randomisation. As rucaparib support was ceased, this futility analysis would form the primary analysis of this treatment arm comparison. Data were analysed using Stata version 17.0 (StataCorp, College Station, TX).

### Tissue sample collection and preparation

Archival formalin fixed paraffin embedded (FFPE) tumour samples were collected at registration. This could be from the initial diagnostic biopsies of advanced/metastatic disease or from surgical specimens for those who relapsed after previous resections. FFPE blocks were sectioned for haematoxylin–eosin (H&E) and 5 × 10 μm sections. DNA was extracted from macrodissected FFPE sections using the Promega Maxwell RSC FFPE Plus DNA Kit on the Maxwell RSC 48 Instrument. DNA quality was assessed using the QUBIT® 3 fluorometer (Invitrogen, Carlsbad, CA). DNA libraries were prepared using the Roche KAPA HyperPlus Kit (Roche Sequencing, Basel, Switzerland) with IDT UDI 8bp adapters (Integrated DNA Technologies, Coralville, IA) and dual-SPRI size selection of the libraries (300-500 bp). Between 50 and 200 ng of input DNA was used per library.

For this translational analysis, samples with ≥20% tumour content were eligible for next-generation sequencing (NGS), and those with ≥30% tumour content were eligible for HRD analysis.

### HRD assessment

HRD was evaluated using a commercial pipeline, the SOPHiA genetics HRD solution (GIInger Genomic Integrity), originally developed and validated in ovarian cancer,^36^ in an ISO 15189:2022-accredited laboratory (laboratory number 20653). Pooled libraries were sequenced using low pass whole genome sequencing (lpWGS; ∼1 × coverage) on the NovaSeq6000 platform (Illumina, San Diego, CA) with 100 bp paired-end reads. Demultiplexing of raw data was carried out using Dragen BCL Convert, and FASTQ files were uploaded to the SOPHiA DDM™ platform for analysis using the GIInger™ deep-learning algorithm.

The SOPHiA GIInger™ solution demonstrated high analytical concordance with the Myriad myChoice HRD assay, which served as the reference assay for the standard of care HRD testing at The Royal Marsden for ovarian cancer.^37^ Owing to the absence of a validated HRD assay for OG cancer, and given the local availability, this platform was selected for our exploratory analysis.

Tumours were classified according to the genomic integrity score (GIS), ranging from –20 to +20. A GIS of ≥0 defined HRD-positive tumours, while scores of <0 were classified as HRD-negative. Medium quality samples for which the deep-learning algorithm did not succeed in computing a GIS due to insufficient signal-to-noise ratio were classified as HRD-inconclusive, commonly due to insufficient tumour content. This assay evaluates HRD exclusively through genome-wide instability derived from lpWGS and does not incorporate tumour *BRCA* mutation status unlike other assays.

### Assessment of somatic genes

NGS was carried out using the RMH200 solid tumour capture panel of 233 genes (Royal Marsden Hospital, London, UK) which includes key somatic DDR genes such as *BRCA1* and *BRCA2* (Supplementary Table S1, available at https://doi.org/10.1016/j.esmoop.2026.108315). Pooled libraries (1 μg) were hybridised to the custom capture panel and sequenced to a mean depth of 400× on the NovaSeq6000 platform (Illumina) with 100 bp paired-end reads. Variants were classified using an automated tier-based system in accordance with the American College of Medical Genetics and Genomics consensus guidelines.^38^ Only pathogenic and likely pathogenic somatic variants (tier 1: strong clinical significance, tier 2: potential clinical significance) were included in analyses.

Results were visualised using oncoplots generated in Python (pyoncoprint v0.2.5) and were ordered by study arm (rucaparib or surveillance), response, and HRD status. Since no radiological responses were observed, PFS was used as the response metric: non-responder (<3.4 months), responder (≥3.4 to 9 months), and good responder (>9 months). The 3.4-month cut-off corresponded to the median PFS across both arms. The 9-month cut-off was selected to identify patients with particularly durable benefit, based on the approximate average PFS among the best responders. These groupings were intended for visual interpretation only and were not predefined or statistically optimised thresholds.

### Study oversight

PLATFORM was an academic study sponsored by The Royal Marsden NHS Foundation Trust. The study adhered to the principles of the International Ethical Guidelines for Biomedical Research Involving Human Subjects, the Declaration of Helsinki, and the International Council for Harmonisation Good Clinical Practice guidelines. The protocol received approval from the National Research Ethics Committee London Southeast (14/LO/1206) and the Medicines and Healthcare Products Regulatory Agency (Endura CT Number: 2014-002169-30). Trial oversight was maintained by the sponsor, investigators, and an independent data monitoring committee. The trial is registered at ClinicalTrials.gov (NCT02678182).

## Results

### Patients

**Figure 1:**
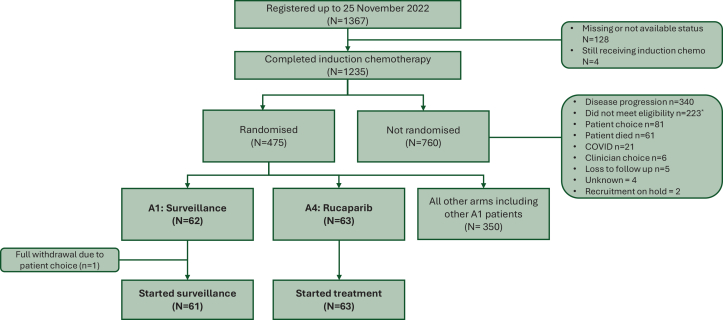
**CONSORT diagram.** Patients were randomly assigned contemporaneously to surveillance or maintenance rucaparib. ∗Patients did not meet eligibility because of a variety of reasons. Most common reasons included: meeting whole trial exclusion criteria such as autoimmune disease/tuberculosis/thromboembolism; not completing full cycles (18 weeks) of chemotherapy; inability to be randomly assigned/to start study treatment on time following induction chemotherapy; receiving interim treatment such as radiotherapy/surgery.

Between March 2015 and November 2022, 1367 patients were registered within the entire study, 1235 completed induction chemotherapy, and 475 patients were randomised. Disease progression was the most common reason for non-randomisation. Between November 2017 and November 2022, 62 patients were contemporaneously randomised to surveillance and 63 patients to maintenance rucaparib, forming the intention-to-treat (ITT) population of 125 patients. One surveillance patient fully withdrew after randomisation, resulting in a safety population of 124 patients (surveillance: 61, rucaparib: 63) (Figure 1). During treatment/surveillance, 13 patients withdrew from the study across both arms. Of these, some withdrew partially, meaning that treatment or surveillance reviews ceased but survival data continued to be collected, while others withdrew fully, meaning that all protocol-specified visits discontinued, including survival follow-up. In addition, one patient in the surveillance arm was lost to follow-up (Supplementary Table S2, available at https://doi.org/10.1016/j.esmoop.2026.108315).

Table 1 describes baseline characteristics which were similar between the two groups. More patients in the surveillance arm achieved a complete or partial response (CR/PR) to induction chemotherapy, while more patients in the rucaparib arm had SD.

**Table 1:** Baseline characteristics between surveillance and rucaparib groups

	Surveillance	Rucaparib
	*N* = 62	*N* = 63
Age, median, years (IQR)	64 (55-71)	65 (58-73)
Gender, *n* (%)		
Female	14 (23)	12 (19)
Male	48 (77)	51 (81)
Primary site, *n* (%)		
Oesophagus	21 (34)	20 (32)
OG junction	22 (35)	24 (38)
Stomach	19 (31)	19 (30)
Histology, *n* (%)		
Well differentiated	3 (5)	1 (2)
Moderately differentiated	18 (29)	17 (27)
Poorly differentiated	37 (60)	43 (68)
Not available	4 (6)	2 (3)
ECOG performance status		
0	30 (48)	37 (59)
1	31 (50)	24 (38)
2	1 (2)	2 (3)
Presentation, *n* (%)		
*De novo* metastatic/locally advanced	55 (89)	54 (86)
Relapsed	7 (11)	9 (14)
Extent of disease, *n* (%)		
Locally advanced	4 (6)	7 (11)
Metastatic	58 (94)	56 (89)
Number of metastatic sites, *n* (%)		
≤1	46 (74)	45 (71)
≥2	16 (26)	18 (29)
Liver metastases, *n* (%)		
Metastatic patients only	*n* = 58	*n* = 56
No	39 (67)	34 (61)
Yes	19 (33)	22 (39)
Induction chemotherapy, *n* (%)^a^^,^^b^		
EOX	17 (27)	25 (40)
CAPOX	32 (52)	30 (48)
ECX	21 (16)	18 (13)
FOLFOX	4 (6)	0 (0)
CX	3 (5)	2 (3)
Carbo-X	1 (2)	0 (0)
FLOT	0 (0)	1 (2)
Duration of induction chemotherapy, *n* (%)		
18 weeks	55 (89)	56 (89)
>18 weeks	6 (10)	6 (10)
<18 weeks^a^	1 (2)	1 (2)
Response to induction chemotherapy, *n* (%)		
Complete or partial response	29 (47)	22 (35)
Stable disease	33 (53)	41 (65)

CAPOX, capecitabine/oxaliplatin; Carbo-X, carboplatin/capecitabine; CF, cisplatin/5-fluorouracil; CX, cisplatin/capecitabine; ECOG, Eastern Cooperative Oncology Group; ECX, epirubicin/cisplatin/capecitabine; EOF, epirubicin/oxaliplatin/fluorouracil; EOX, epirubicin/oxaliplatin/capecitabine; FLOT, fluorouracil/leucovorin/oxaliplatin/docetaxel; IQR, interquartile range.

a Protocol version 7 allowed eight cycles of two-weekly treatment and permitted triplet fluoropyrimidine-platinum-based chemotherapy.

b Patients may be included across multiple rows.

### Treatment

At data cut-off on 28 April 2023, one (2%) surveillance and five (8%) rucaparib patients remained on treatment. Disease progression was the most common reason for discontinuation (88% surveillance and 83% rucaparib). In the rucaparib arm, discontinuation also occurred due to two patient deaths and one due to toxicity. The median duration of rucaparib was 2.8 months [interquartile range (IQR) 1.5-5.5]. The median number of rucaparib cycles administered was 4 (range 1-37). Of 63 patients receiving rucaparib, 6 (10%) required at least one dose reduction, and treatment delays occurred in 17 (27%) patients, primarily for toxicity.

At disease progression, more surveillance patients received subsequent treatment compared with patients in the rucaparib arm (44% versus 33% respectively) (Supplementary Table S3, available at https://doi.org/10.1016/j.esmoop.2026.108315).

### Efficacy

At time of analysis, 90% (56/62) of patients in the surveillance and 87% (55/63) of patients in the rucaparib arm had progressed, while 74% (46/62) and 81% (51/63) of patients had died respectively. The median follow-up for the ITT population was 41 months.

**Figure 2:**
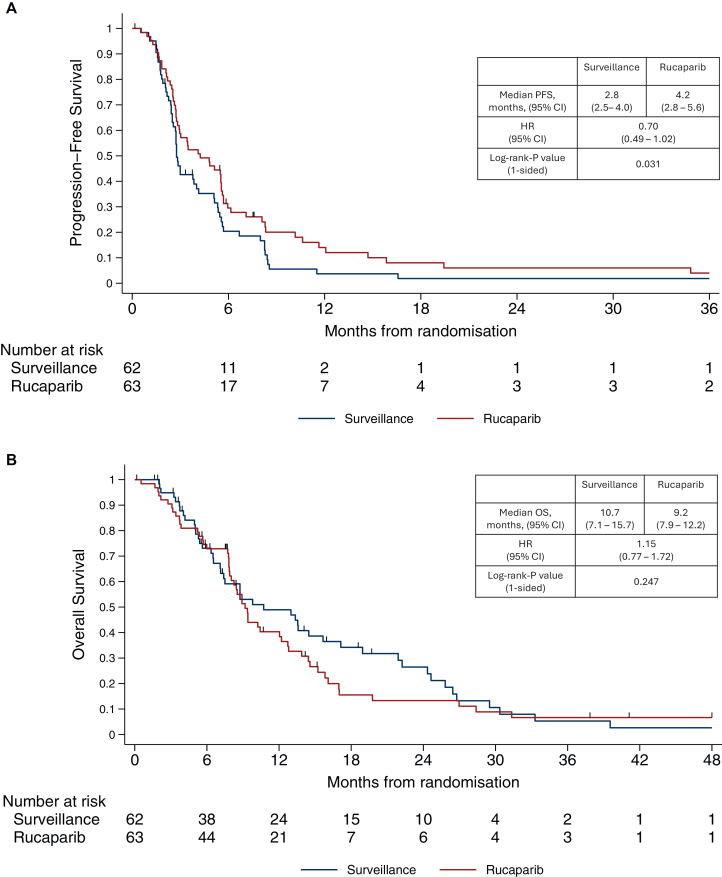
**Survival outcomes in the intention-to-treat population.** (A) PFS from randomisation. (B) OS from randomisation. CI, confidence interval; HR, hazard ratio; OS, overall survival; PFS, progression-free survival.

Figure 2A shows PFS. There was no significant difference between the two arms (unadjusted HR 0.70, 95% CI 0.49-1.02, one-sided *P* value = 0.031). The median PFS was 2.8 months (95% CI 2.5-4.0) in the surveillance arm and 4.2 months (95% CI 2.8-5.6) in the rucaparib arm. The 12-month PFS rate was 3.7% and 14.0%, and the 24-month PFS rate was 1.9% and 6.0%, respectively. PFS subgroup analysis suggested that patients with ECOG PS 1-2, poorly differentiated tumours, had CR/PR to first-line chemotherapy, metastatic, and primary tumours in the oesophagus and GOJ appeared to derive greater benefit (Supplementary Figure S2, available at https://doi.org/10.1016/j.esmoop.2026.108315) from rucaparib.

Figure 2B shows OS. No OS difference was observed between the two arms (unadjusted HR 1.15, 95% CI 0.77-1.72, one-sided *P* value = 0.247). The median OS was 10.7 months in the surveillance arm and 9.2 months in the rucaparib arm. At 12 months, the OS rate was 48.9% for surveillance and 40.3% for rucaparib, and at 24 months, 26.5% surveillance and 13.3% rucaparib patients were alive, respectively.

Within the modified ITT (mITT) population, the 12-week PFR was 36% (95% CI 24-49) in the surveillance arm and 55% (95% CI 42-68) in the rucaparib arm (odds ratio 2.2, 95% CI 1.1-4.6, one-sided *P* value = 0.018). This met the interim analysis for non-futility and would have supported continuing recruitment to full accrual. No radiological responses by RECIST were seen in either arm and therefore the ORR endpoint was not evaluated. However, only 39% (23/59) of patients in the surveillance arm and 39% (24/62) of patients in the rucaparib arm from the mITT had measurable disease at randomisation.

Thirty-four patients had SD in the rucaparib arm at 3 months as no CR/PR was observed with rucaparib. Of these 34 patients, 20 (59%) had SD to first-line chemotherapy, whereas 14 (41%) had PR/CR. Furthermore, of the 63 patients treated with rucaparib, we observed better PFS with the patients who had CR/PR to first-line chemotherapy (unadjusted HR 0.62, 95% CI 0.35-1.09). Supplementary Figure S3, available at https://doi.org/10.1016/j.esmoop.2026.108315 shows PFS in the rucaparib arm according to response to first-line chemotherapy.

### HRD and survival outcomes

A total of 41% (51/125) patients were eligible for HRD analysis (surveillance: 20, rucaparib: 31). Just over 50% of patients with available FFPE samples were excluded from analysis; 30% did not meet the HRD assay tumour content requirement of ≥30%, and 23% had poor quality DNA so were not eligible for analysis (Supplementary Figure S4, available at https://doi.org/10.1016/j.esmoop.2026.108315). Across the cohort of 51 patients, 5 (10%) patients were HRD-positive, 41 (80%) HRD-negative, and 5 (10%) had inconclusive HRD results. Among the HRD-positive group, four patients were treated with rucaparib and had PFS of 2.6, 3.0, 8.3, and 10.6 months, respectively. The single HRD-positive patient in the surveillance arm had a PFS of 1.8 months. Supplementary Figure S5, available at https://doi.org/10.1016/j.esmoop.2026.108315 shows PFS and OS by HRD status between each arm; however, comparisons are not possible given the small subgroup size.

### Somatic gene assessment and PFS outcome

Of the 125 patients enrolled, tumour samples from 42% (53/125) were of sufficient quality for NGS (surveillance: *n* = 19, rucaparib: *n* = 34). At least one somatic alteration was detected in 53% (28/53) of patients (Supplementary Figures S6 and S7, available at https://doi.org/10.1016/j.esmoop.2026.108315). *TP53* was the most frequently mutated gene (82%), predominantly missense variants, followed by *PIK3CA* (18%), *CDKN2A* (18%), *SMARCA4* (14%), and *ARID1A* (14%). Several of these genes are functionally linked to DDR pathways, suggesting a DDR-associated genomic phenotype in a subset of tumours. However, no clear association with PFS was observed. The distribution of somatic alterations was similar between the rucaparib and surveillance arms, and no patients in the surveillance arm had PFS >9 months.

Alterations in key homologous recombination repair (HRR) genes were rare, with *BRCA2* mutations identified in three patients through tumour-only sequencing; germline status was not available. One patient in the rucaparib arm was a non-responder, and the remaining two patients, both in the surveillance arm, were also categorised as non-responders with PFS <3.4 months. Notably, the longest responder in the rucaparib arm remained progression-free at 38 months and was classified as HRD-negative, harbouring somatic alterations in *TP53*, *PIK3CA,* and *CDKN2A* (Supplementary Figure S6, available at https://doi.org/10.1016/j.esmoop.2026.108315 patient 17).

Among the five HRD-positive patients, three had detectable somatic alterations. Two received rucaparib; one achieved prolonged PFS (10.6 months) with a *PIK3CA* missense mutation, while the other progressed early (3.0 months) despite harbouring DDR-associated alterations in *BRCA2*, *SMARCA4*, *TP53*, and *PTEN*. The remaining third HRD-positive patient in the surveillance arm had short PFS (1.8 months), with *SMARCA4* and *BRCA2* (Supplementary Table S4, available at https://doi.org/10.1016/j.esmoop.2026.108315).

### Safety

Table 2 shows adverse events (AEs) in the safety population (*n* = 124). AEs were reported in 98% of patients in both groups. The frequency of grade 1 and 2 AEs was similar between arms, occurring in 75% of patients in the surveillance arm and 67% of patients in the rucaparib arm. Grade ≥3 AEs were more frequent in the rucaparib arm (32%) compared with surveillance (23%). Treatment-related AEs (trAEs), defined as events possibly or probably related to rucaparib, were reported in 84% (53/63) of patients, which were deemed grade 1 (38%), grade 2 (40%), grade 3 (17%), and grade 4 (6%) events. The most common grade 3-4 trAEs were anaemia, fatigue, infection, and neutropenia. Six serious adverse events (SAEs) occurred in the surveillance arm, most commonly dysphagia and infection. In comparison, 16 SAEs were reported in the rucaparib arm, primarily anaemia, dysphagia, and infection, with anaemia representing the most frequent grade 3-4 SAE. No treatment-related deaths were observed.

**Table 2:** AEs in the safety population

	Surveillance (*N* = 61)		Rucaparib (*N* = 63)	
	Any grade	Grade ≥ 3	Any grade	Grade ≥ 3
**AE, *n* (%)**	60 (98)	14 (23)	62 (98)	20 (32)
Related to rucaparib	-	-	53 (84)	14 (22)
**AE in ≥10% of patients in any arm, *n* (%)**				
Anaemia	13 (21)	0 (0)	31 (49)	2 (3)
Anorexia	13 (21)	0 (0)	26 (41)	1 (2)
Anxiety	10 (16)	0 (0)	12 (19)	1 (2)
Constipation	10 (16)	0 (0)	26 (41)	0 (0)
Cough	11 (18)	0 (0)	18 (29)	1 (2)
Depression	7 (11)	0 (0)	7 (11)	1 (2)
Diarrhoea	10 (16)	0 (0)	13 (21)	0 (0)
Dizziness	10 (16)	0 (0)	10 (16)	0 (0)
Dysgeusia	4 (7)	0 (0)	11 (17)	0 (0)
Dysphagia	15 (25)	2 (3)	19 (30)	4 (6)
Dyspnoea	12 (20)	0 (0)	17 (27)	1 (2)
Fatigue	29 (48)	0 (0)	44 (70)	4 (6)
Fever	5 (8)	0 (0)	8 (13)	1 (2)
Hypertension	7 (11)	1 (2)	11 (17)	1 (2)
Infection	12 (20)	0 (0)	15 (24)	3 (5)
Insomnia	5 (8)	0 (0)	8 (13)	0 (0)
Mucositis	2 (3)	0 (0)	7 (11)	0 (0)
Nausea	11 (18)	0 (0)	27 (43)	0 (0)
Neutropenia	3 (5)	0 (0)	6 (10)	1 (2)
Pain	33 (54)	2 (3)	39 (62)	2 (3)
Peripheral neuropathy	37 (61)	0 (0)	47 (75)	1 (2)
Plantar-palmar erythema	6 (10)	0 (0)	14 (22)	0 (0)
Raised ALT	8 (13)	0 (0)	17 (27)	1 (2)
Raised AST	7 (11)	0 (0)	16 (25)	0 (0)
Reflux	9 (15)	0 (0)	8 (13)	0 (0)
Thrombocytopenia	5 (8)	0 (0)	9 (14)	0 (0)
Vomiting	8 (13)	1 (2)	18 (29)	2 (3)

AE, adverse events; ALT, alanine aminotransferase; AST, aspartate aminotransferase.

## Discussion

In this analysis, maintenance rucaparib was compared with surveillance in patients with HER2-negative advanced OGA who completed first-line platinum-based chemotherapy. While the interim 12-week PFR results supported continuation to full accrual, recruitment closed prematurely following withdrawal of industry support. Consequently, fewer than half of the required PFS events occurred, rendering the study underpowered for its primary endpoint. Median PFS was numerically longer with maintenance rucaparib than with surveillance (4.2 versus 2.8 months; HR 0.70, *P* = 0.031), but this difference was not statistically significant.

No difference in OS was observed, with numerically longer OS in the surveillance arm. This finding is likely explained by imbalances in post-progression therapies, as a greater proportion of patients in the surveillance arm received subsequent treatment. This may reflect reduced tolerance to further therapy among patients who received rucaparib due to fatigue or myelosuppression. An alternative explanation is the emergence of acquired resistance to PARP inhibition. For example, in ovarian, prostate, and pancreatic cancers, studies have identified reversion variants that restore HRR function, which are associated with resistance to both PARP inhibitors and platinum-based chemotherapy and may contribute to poorer subsequent outcomes.^39^, ^40^, ^41^ No incremental radiological responses were observed with rucaparib, although fewer than half of patients had measurable disease at randomisation, and in the maintenance setting most responses would have arisen during first-line chemotherapy. Rucaparib was generally well tolerated and aligned with prior experience of PARP inhibitors across other tumour types.^20^^,^^25^^,^^26^^,^^30^^,^^42^ Key limitations include early trial closure and a small sample size, which restrict full assessment of rucaparib efficacy.

Several studies of PARP inhibitors in advanced OGA have been conducted. In the phase II study 39 trial, olaparib plus paclitaxel did not meet the endpoint of improving PFS compared with placebo plus paclitaxel, although an OS benefit was observed, particularly in the prospectively enriched ataxia telangiectasia mutated (ATM)-low subgroup (overall population: HR 0.56; *P* = 0.005; ATM-low: HR 0.35, *P* = 0.002).^43^ These findings were not confirmed in the subsequent phase III GOLD trial, which just failed to meet its OS endpoints in either the overall or ATM-low populations, potentially reflecting the lower prevalence of ATM-low tumours due to lack of biomarker enrichment.^44^ Together, these findings suggest that any activity of PARP inhibitors in OGA may be limited to biomarker-selected populations enriched for HRD, although evidence remains inconclusive.

In the maintenance setting, results from the phase II PARALLEL-303 trial align with our findings, showing a numerically longer but non-significant PFS with pamiparib compared with placebo (3.7 versus 2.1 months; HR 0.8, *P* = 0.1428) with OS longer in the placebo group as well.^42^ Collectively, these results suggest that maintenance PARP inhibition may modestly delay disease progression following platinum-fluoropyrimidine chemotherapy in advanced OGA, although clear clinical benefit remains unproven. It is possible that rucaparib was not the most optimal PARP inhibitor in advanced OGA and other novel PARP or PARG [poly(ADP-ribose) glycohydrolase] inhibitors may have better efficacy shown in future trials.

The phase III ARMANI trial, which evaluated paclitaxel-ramucirumab (pac-ram) as early switch maintenance after 3 months of oxaliplatin-based induction doublet chemotherapy against continuation of chemotherapy followed by fluoropyrimidine maintenance in 280 patients with advanced HER2-negative gastric or gastroesophageal junction cancers, showed significant prolongation of PFS by 3.1 months (HR 0.61, *P* = 0.0002) and OS by 2.2 months (HR 0.75, *P* = 0.025) albeit with significantly higher grade ≥3 trAEs.^45^ The purpose of maintenance therapy is to not only sustain disease control but more importantly maintain quality of life and allow recovery from toxicities secondary to platinum-fluoropyrimidine induction chemotherapy, namely peripheral neuropathy and myelosuppression. It seems intuitive that alternating therapies in chemotherapy responders would prolong survival, but early introduction of pac-ram should not come at the cost of maximum benefit from platinum-based induction chemotherapy, increased toxicity, and more frequent hospital visits. In our PLATFORM study, cape-ram did also show significant PFS (HR 0.33, *P* < 0.001) and OS (HR 0.51, *P* = 0.023) benefit with better tolerance.^33^

Maintenance avelumab was evaluated in the global phase III JAVELIN Gastric 100 study compared with continued chemotherapy in patients without progressive disease after 12 weeks of fluoropyrimidine and oxaliplatin combination chemotherapy.^46^ Taken together with the maintenance durvalumab arm of our PLATFORM study,^34^ the median PFS in the immunotherapy arms of both trials was similar (3.0 months in PLATFORM and 3.2 months in JAVELIN Gastric 100) as were median OS (11.3 and 10.4 months, respectively). The phase II MOONLIGHT trial also reported a lack of benefit of combined PD-1 and CTLA-4 blockade maintenance following 6 weeks of modified FOLFOX chemotherapy with a PFS at 6 months of 30%, in comparison to 48% when chemotherapy was administered in parallel with dual immune checkpoint blockade.^47^ This reinforces the concept that immune checkpoint inhibition is best utilised upfront concurrently with chemotherapy in advanced OGA in a biomarker-selected population and that its use in the maintenance setting does not provide adequate antitumour activity, even in patients with ongoing disease control.

Platinum sensitivity may be a potential biomarker for PARP inhibition efficacy in OGA. Whereas we observed numerically better PFS for patients who achieved CR/PR to first-line platinum-based chemotherapy when treated with rucaparib (Supplementary Figures S2 and S3, available at https://doi.org/10.1016/j.esmoop.2026.108315), the upper limit of 95% CI crossed unity; thus, no statistical significance could be inferred. In addition, for patients with more prolonged disease control with rucaparib, no clear trend was shown to platinum sensitivity to first-line chemotherapy. To further explore potential biomarkers of response, HRD assessment and NGS were carried out based on the biological premise that defects in homologous recombination and related DNA repair pathways may confer sensitivity to PARP inhibition. However, HRD remains poorly characterised in OGA. Prior studies estimate that alterations in DDR pathways occur in ∼7%-12% of gastric cancers and 18% of oesophageal cancers.^48^ In the REAL-3 trial, HRD was assessed using a genomic loss-of-heterozygosity (LOH) signature. LOH-high was identified in 14% of patients and was associated with improved survival, although this analysis was limited by tissue attrition and small numbers.^49^ Alternative measures of DNA repair deficiency have been explored. A 44-gene transcriptional DNA damage immune response signature was developed in breast cancer.^50^^,^^51^ This signature was subsequently used to identify subsets of OGA patients with improved responses to platinum-based chemotherapy in both curative and advanced settings.^52^^,^^53^ Reported prevalence varies from ∼12% to 24%, highlighting heterogeneity depending on disease context and methodology.^52^^,^^53^

Utilising our HRD analysis methodology with SOPHiA GIInger™ solution, HRD was identified in 10% of patients with evaluable tumour samples, broadly consistent with previous estimates. However, limited sample size and the exploratory nature of the analysis precluded robust assessment of associations between HRD status and clinical outcomes. Overall, HRD positivity did not predict clinical benefit from maintenance rucaparib. Among HRD-positive patients treated with rucaparib, PFS outcomes were heterogenous, ranging from early progression to prolonged disease control. Conversely, the longest responder in the rucaparib arm was HRD-negative and harboured somatic alterations in *TP53*, *PIK3CA,* and *CDKN2A*. Although based on a single patient, this finding raises the possibility that PARP inhibitor sensitivity may occur outside conventional genomic definitions of HRD. Similar hypotheses have been proposed in other tumour types, describing a broader “BRCAness” phenotype in which functional HRD arises through alternative biological mechanisms.^54^^,^^55^

*TP53* was the most frequently altered gene, consistent with its known prevalence in OGA and its central role in genomic stability.^11^^,^^56^ Alterations in chromatin remodelling genes such as *ARID1A* and *SMARCA4* were also common. While loss of *ARID1A* has been linked to PARP inhibitor sensitivity in other cancers, no clear correlation with improved PFS was observed in this cohort, highlighting the limitations of single-gene alterations as predictive biomarkers in isolation.^57^^,^^58^ Core HRR gene alterations were rare, and *BRCA2* mutations were not associated with benefit. Janjgian et al prospectively sequenced 295 patients with metastatic gastric cancer and found no significant association between individual DDR gene alterations or LST score, used as a surrogate measure of HRD, and improved PFS response following first-line platinum-based chemotherapy.^59^ These findings highlight the complexity of translating genomic measures of DNA repair deficiency into clinically actionable biomarkers in OGA.

A major limitation of the translational analysis was substantial tumour sample attrition, with over half of FFPE specimens excluded due to inadequate tumour content or poor DNA quality. The use of archival rather than fresh tissue limited data quality, and prior translational work within the maintenance durvalumab arm of PLATFORM exhausted some surveillance samples.^34^ These challenges are common in advanced OGA, where diagnostic material is often limited to small biopsies. Consequently, statistical power was insufficient for formal comparisons and results should be considered descriptive. Despite availability of archival tissue was a mandatory requirement in our PLATFORM eligibility criteria, even after sample collection, during the conduct of the study, the submitted archival tissue could be requested back from investigator sites for other clinical purposes before we carried out our translational analyses within the study. Furthermore, it was an optional consent for genetic testing which would have contributed to further sample attrition.

In our study, archival tissue was obtained from initial diagnostic biopsies or previous resection specimens at trial registration without further fresh biopsies at the time of randomisation. This would hamper any assessment of potential resistance mechanisms to PARP inhibitors, acquired during the initial first-line platinum-based chemotherapy. Heterogeneity could also be present for surgical specimens if they had received perioperative platinum-based chemotherapy. Indeed, of the 62 surveillance patients, 7 had presented with relapsed disease. Of these, four patients had previous preoperative chemotherapy and four had previous post-operative chemotherapy. Of the 63 patients in rucaparib, 9 had presented with relapsed disease. Of these, six patients had previous preoperative chemotherapy and four patients had previous post-operative chemotherapy. However, we did not collect any details on perioperative chemotherapy regimens, although the vast majority would be expected to have a platinum-based regimen.

An additional limitation is the absence of a validated HRD assay for OGA. The HRD-SOPHiA assay used here has been validated in ovarian cancer, where HRD-associated genomic scars are well characterised. However, tumour-specific differences in mutational drivers and genomic instability may affect assay performance and interpretation in OGA. As such, HRD status as defined here should be regarded as exploratory and hypothesis generating. Independent datasets might clarify whether a specific HRD assay is required in OGA and such data may be forthcoming from our second-line olaparib SOLAR study.^60^

### Conclusion

Maintenance rucaparib did not significantly improve PFS compared with surveillance in patients with advanced HER2-negative OGA controlled on first-line chemotherapy. Exploratory translational analyses provided preliminary insight into the prevalence of HRD in this population but overall, HRD status and individual gene alterations did not consistently correlate with PFS benefit.
