# Supplementary material for: Maintenance rucaparib after first-line platinum-based chemotherapy in HER2-negative advanced oesophagogastric adenocarcinoma: results from the PLATFORM study
Source: ESMO Open. 2026 Aug 10;11(9):108315. doi: 10.1016/j.esmoop.2026.108315 (PMC13486810; doi:10.1016/j.esmoop.2026.108315)
Supplement: Supplementary file 1 [file mmc3.pdf]

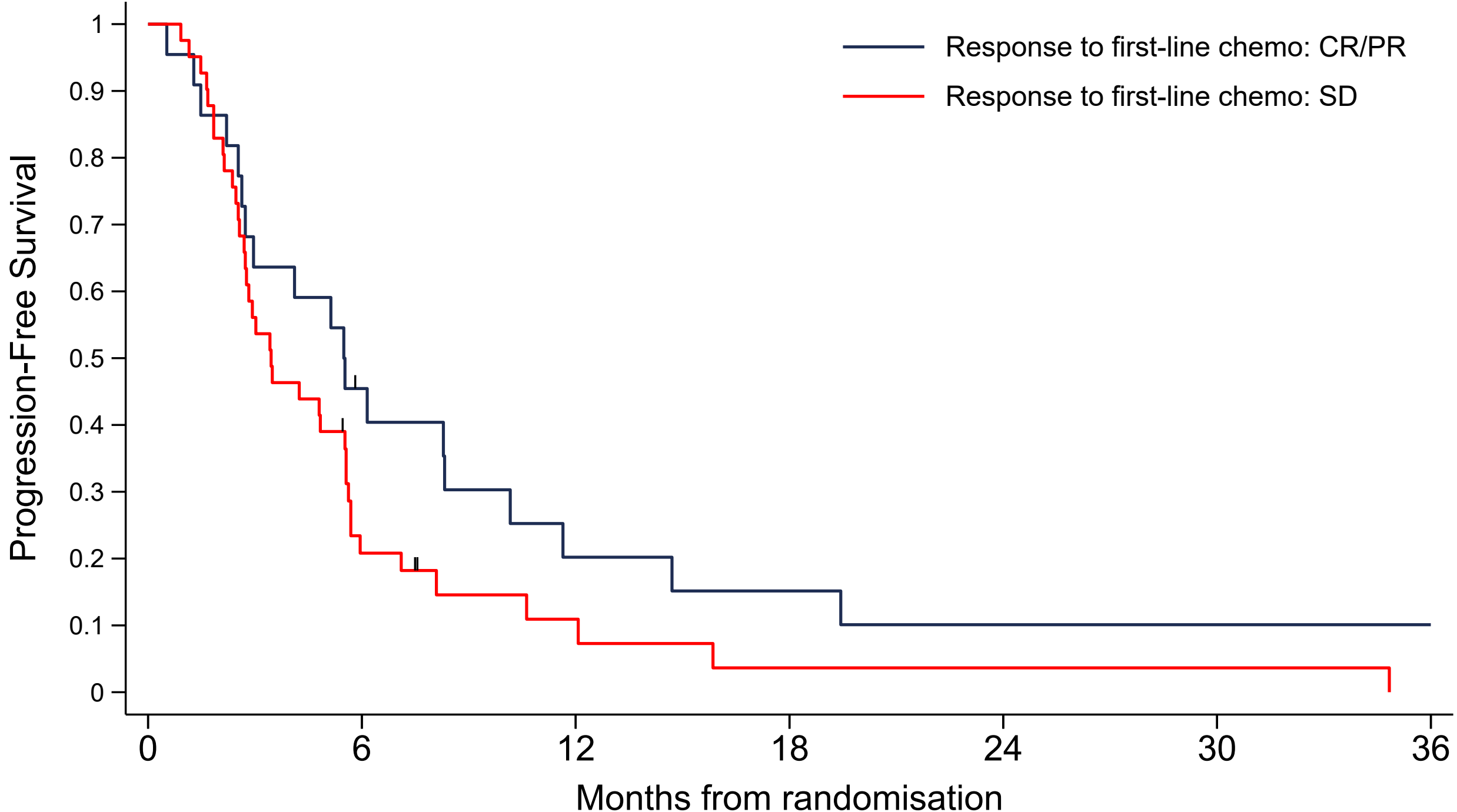

Number at risk

CR/PR	22	9	4	3	2	2	2
SD	41	8	3	1	1	1	0
