# Supplementary material for: Maintenance rucaparib after first-line platinum-based chemotherapy in HER2-negative advanced oesophagogastric adenocarcinoma: results from the PLATFORM study
Source: ESMO Open. 2026 Aug 10;11(9):108315. doi: 10.1016/j.esmoop.2026.108315 (PMC13486810; doi:10.1016/j.esmoop.2026.108315)
Supplement: Supplementary file 2 [file mmc10.docx]

**Supplementary table 4:** Characteristics of the five HRD-positive patients across the cohort

| Patient | Trial Arm | Primary tumour location | Response to first-line chemotherapy | Best RECIST response on trial | PFS, months | Somatic alterations detected |
| --- | --- | --- | --- | --- | --- | --- |
| 18 | Surveillance | GOJ | PR | PD | 1.7 | SMARCA4, BRCA2, TET2 |
| 29 | Rucaparib | Stomach | PR | SD | 2.6 |  |
| 30 | Rucaparib | GOJ | PR | SD | 8.3 |  |
| 14 | Rucaparib | GOJ | SD | SD | 10.6 | PIK3CA |
| 1 | Rucaparib | Oesophagus | PR | PD | 3.0 | SMARCA4, BRCA2, PTEN, TP53 |

Legend: GOJ – gastroesophageal junction, PFS – progression-free survival, PR – partial response, SD – stable disease,
