# Supplementary material for: Maintenance rucaparib after first-line platinum-based chemotherapy in HER2-negative advanced oesophagogastric adenocarcinoma: results from the PLATFORM study
Source: ESMO Open. 2026 Aug 10;11(9):108315. doi: 10.1016/j.esmoop.2026.108315 (PMC13486810; doi:10.1016/j.esmoop.2026.108315)
Supplement: Supplementary file 3 [file mmc11.docx]

**Supplementary table 3:** Post progression therapies

|  | Surveillance  N=61 | Rucaparib  N=63 |
| --- | --- | --- |
| Number of patients started radiotherapy, n (%) | 13 (21) | 15 (24) |
| Number of patients undergone surgery, n (%) | 4 (7) | 8 (13) |
| Number of patients having 2^nd^/3^rd^/4^th^ line subsequent chemotherapy, n (%) | 27 (44) | 21 (33) |
| Number of patients having 3^rd^/4^th^ line, n (%) | 14 (23) | 5 (8) |
