# Supplementary material for: Maintenance rucaparib after first-line platinum-based chemotherapy in HER2-negative advanced oesophagogastric adenocarcinoma: results from the PLATFORM study
Source: ESMO Open. 2026 Aug 10;11(9):108315. doi: 10.1016/j.esmoop.2026.108315 (PMC13486810; doi:10.1016/j.esmoop.2026.108315)
Supplement: Supplementary file 4 [file mmc8.docx]

**Supplementary table 1:** RMH200Solid gene panel

| **RMH200v2** | **Regions of interest** | **Additional features** |
| --- | --- | --- |
| ABL1 | x4,5,6,7 | MSI |
| ACVR1 | 3 to 11 | QC SNP |
| AKT1 | all; CNV | CNV (1Mb resolution) |
| AKT2 | exon3 (E17K) + CNV |  |
| AKT3 | exon3 (E17K) |  |
| ALK | 19 to 29 +CNV |  |
| AMER1 | all |  |
| ANTXR2 | all |  |
| APC | all |  |
| AR | all |  |
| ARAF | all |  |
| ARID1A | all |  |
| ARID1B | all |  |
| ARID2 | all |  |
| ASXL1 | all; CNV |  |
| ATM | all; CNV |  |
| ATR | all |  |
| ATRX | all; CNV |  |
| AURKA | all; CNV |  |
| AXIN1 | all |  |
| AXIN2 | all |  |
| B2M | all |  |
| BAP1 | all |  |
| BARD1 | all |  |
| BBC3 | all |  |
| BCL2 | all |  |
| BCOR | all; CNV |  |
| BCORL1 | all |  |
| BIRC3 | all; CNV |  |
| BRAF | all (inc introns8910 tx); CNV |  |
| BRCA1 | all |  |
| BRCA2 | all |  |
| BRIP1 | all |  |
| BTK | exon 15 (C481S) |  |
| C19MC | all; CNV |  |
| CALR | exon 9 |  |
| CASP8 | all |  |
| CBL | ex8,9 |  |
| CCND1 | all; CNV |  |
| CCND2 | all; CNV |  |
| CCND3 | CNV only |  |
| CCNE1 | all; CNV |  |
| CCNE2 | all; CNV |  |
| CD79b | all; CNV |  |
| CDH1 | all; CNV |  |
| CDK12 | all |  |
| CDK2 | all |  |
| CDK4 | all; CNV |  |
| CDK6 | all; CNV |  |
| CDKN1A | all |  |
| CDKN2A | all; CNV |  |
| CDKN2B | all; CNV |  |
| CDKN2C | all; CNV |  |
| CEBPA | all; CNV |  |
| CHEK1 | all; CNV |  |
| CHEK2 | all |  |
| CIC | all |  |
| CDKN1B | all; CNV |  |
| CKS1B | all; CNV |  |
| CREBBP | all |  |
| CRLF1 | CNV only |  |
| CTNNB1 | all |  |
| DAXX | all |  |
| DDR2 | CNV only |  |
| DDX3X | all |  |
| DICER1 | all |  |
| DPYD (hotspots) | all |  |
| DROSHA | all |  |
| EGFR | all; CNV |  |
| EMSY | all |  |
| EP300 | all; CNV |  |
| EPHB2 | all |  |
| ERBB2 | all; CNV |  |
| ERBB3 | all; CNV |  |
| ESR1 | all |  |
| ETV6 | all; CNV |  |
| EZH2 | all; CNV |  |
| F2R | all; CNV |  |
| FADD | all; CNV |  |
| FANCI | all |  |
| FANCL | all |  |
| FAT1 | all |  |
| FBXW7 | all; CNV |  |
| FGF10 | all; CNV |  |
| FGFR1 | all; CNV |  |
| FGFR2 | all; CNV |  |
| FGFR3 | all; CNV |  |
| FGFR4 | all; CNV |  |
| FH | all |  |
| FLT3 | all; CNV |  |
| FOXL2 | all; CNV |  |
| FOXO1 | all; CNV |  |
| GATA1 | all; CNV |  |
| GATA3 | all; CNV |  |
| GNA11 | exon 5 |  |
| GNAQ | exon 5 |  |
| GNAS | exon 5 |  |
| GPR161 | all |  |
| H3F3A | all |  |
| H3F3B | all |  |
| HIST1H3B | all |  |
| HIST1H3C | all |  |
| HIST2H3C | all |  |
| HRAS | all |  |
| ID3 | all |  |
| IDH1 | all; CNV |  |
| IDH2 | all; CNV |  |
| IGF1R | all; CNV |  |
| IRF4 | all; CNV |  |
| IRS2 | all |  |
| JAK2 | ex14 V617 |  |
| KBTBD4 | all |  |
| KDM6A | all |  |
| KDR | ex6-26 |  |
| KEAP1 | all |  |
| KIAA1549 | CNV only |  |
| KIT | all; CNV |  |
| KLF2 | all |  |
| KMT2A | all; CNV |  |
| KMT2C | all; CNV |  |
| KMT2D | all; CNV |  |
| KRAS | all; CNV |  |
| LIN28B | all; CNV |  |
| LZTR1 | all |  |
| MAP2K1 | all |  |
| MAP2K2 | all |  |
| MAP2K4 | all |  |
| MAP3K1 | all |  |
| MAPK1 | all |  |
| MCL1 | all |  |
| MDM2 | all; CNV |  |
| MEN1 | all |  |
| MET | all; CNV |  |
| MLH1 | all |  |
| MN1 | all |  |
| MPL | ex10 |  |
| MRE11A | all |  |
| MSH2 | all |  |
| MSH6 | all |  |
| MTOR | exons 28-58 |  |
| MYC | all; CNV |  |
| MYCL1 | all; CNV |  |
| MYCN | all; CNV |  |
| MYD88 | exon 5 (L265) |  |
| NF1 | all; CNV |  |
| NF2 | all |  |
| NFE2 | all; CNV |  |
| NFE2L2 | all |  |
| NOTCH1 | all |  |
| NOTCH2 | exon 1,2,5,20,30; CNV |  |
| NOTCH3 | all |  |
| NPM1 | exon 10, 11; CNV |  |
| NRAS | all; CNV |  |
| NTRK1 | ex8,9,10 |  |
| NTRK2 | ex17,18 |  |
| NTRK3 | ex16,17 |  |
| OTX1 | all |  |
| PALB2 | all; CNV |  |
| PAX5 | all; CNV |  |
| PBRM1 | all |  |
| PDCD1LG2 | all; CNV |  |
| PDGFRA | all; CNV |  |
| PHOX2B | all |  |
| PIK3CA | all |  |
| PIK3CD | all |  |
| PIK3R1 | all |  |
| PIN1 | all |  |
| PMS1 | all |  |
| PMS2 | all |  |
| POLD1 | all |  |
| POLE | all |  |
| POT1 | all |  |
| PPM1D | all |  |
| PPP2R2A | all |  |
| PRKAR1A | all |  |
| PTCH1 | all |  |
| PTCH2 | all |  |
| PTEN | all; CNV |  |
| PTPN11 | all; CNV |  |
| RAD21 | all; CNV |  |
| RAD50 | all |  |
| RAD51B | all |  |
| RAD51C | all |  |
| RAD51D | all |  |
| RAD54L | all |  |
| RAF1 | all |  |
| RB1 | all; CNV |  |
| RBM10 | all |  |
| RET | all |  |
| RFN43 | all |  |
| RHOA | all |  |
| RICTOR | CNV only |  |
| RIT1 | all |  |
| ROS1 | exons 32-43 |  |
| RUNX1 | all; CNV |  |
| SDHA | all |  |
| SDHB | all |  |
| SDHC | all |  |
| SDHD | all |  |
| SETBP1 | all; CNV |  |
| SETD2 | all |  |
| SF3B1 | all; CNV |  |
| SH2B3 | all |  |
| SMAD2 | all; CNV |  |
| SMAD3 | all |  |
| SMAD4 | all |  |
| SMARCA4 | all |  |
| SMARCB1 | all |  |
| SMARCE1 | all |  |
| SMO | all |  |
| SOX2 | all; CNV |  |
| SRSF2 | all; CNV |  |
| STAG2 | all; CNV |  |
| STK11 | all |  |
| SUFU | all |  |
| TCEB1 | all |  |
| TCF3 | all |  |
| TERT | all; CNV; promoter (-800) |  |
| TET2 | all; CNV |  |
| TFE3 | all |  |
| TG | CNV only |  |
