# Supplementary material for: Maintenance rucaparib after first-line platinum-based chemotherapy in HER2-negative advanced oesophagogastric adenocarcinoma: results from the PLATFORM study
Source: ESMO Open. 2026 Aug 10;11(9):108315. doi: 10.1016/j.esmoop.2026.108315 (PMC13486810; doi:10.1016/j.esmoop.2026.108315)
Supplement: Supplementary file 5 [file mmc9.docx]

**Supplementary table 2:** Withdrawals and loss to follow up frequencies

|  | Surveillance  N=62 | | Rucaparib  N=63 | |
| --- | --- | --- | --- | --- |
|  | N | % | N | % |
| **Withdrawn** |  |  |  |  |
| Fully withdrawal | 8 | 13 | 1 | 2 |
| Partial withdrawal | 0 | 0 | 4 | 6 |
| **Withdrawal Reasons:** |  |  |  |  |
| Adverse event/toxicity | 0 | 0 | 1 | 20 |
| Clinician choice | 0 | 0 | 2 | 40 |
| Patient’s choice | 7 | 88 | 2 | 40 |
| Other | 1 | 13 | 0 | 0 |
| **Lost to follow up** | 1 | 2 | 0 | 0 |
